# Restoration of the Majority of the Visual Spectrum by Using Modified *Volvox* Channelrhodopsin-1

**DOI:** 10.1038/mt.2014.81

**Published:** 2014-06-03

**Authors:** Hiroshi Tomita, Eriko Sugano, Namie Murayama, Taku Ozaki, Fumiaki Nishiyama, Kitako Tabata, Maki Takahashi, Takehiko Saito, Makoto Tamai

**Affiliations:** 1 Laboratory of Visual Neuroscience, Department of Chemistry and Bioengineering, Iwate University Graduate School of Engineering, Morioka, Iwate, Japan; 2 Clinical Research, Innovation and Education Center, Tohoku University Hospital, Sendai, Miyagi, Japan; 3 Department of Ophthalmology, Hirosaki University Graduate School of Medicine, Hirosaki, Aomori, Japan; 4 Tohoku University Graduate School of Medicine, Sendai, Miyagi, Japan

## Abstract

We previously showed that blind rats whose vision was restored by gene transfer of *Chlamydomonas* channelrhodopsin-2 (ChR2) could only detect wavelengths less than 540 nm because of the action spectrum of the transgene product. *Volvox*-derived channelrhodopsin-1, VChR1, has a broader spectrum than ChR2. However, the VChR1 protein was mainly localized in the cytoplasm and showed weak ion channel properties when the VChR1 gene was transfected into HEK293 cells. We generated modified *Volvox* channelrhodopsin-1 (mVChR1), which is a chimera of *Volvox* channelrhodopsin-1 and *Chlamydomonas* channelrhodopsin-1 and demonstrated increased plasma membrane integration and dramatic improvement in its channel properties. Under whole-cell patch clamp, mVChR1-expressing cells showed a photo-induced current upon stimulation at 468–640 nm. The evoked currents in mVChR1-expressing cells were ~30 times larger than those in VChR1-expressing cells. Genetically, blind rats expressing mVChR1 via an adeno-associated virus vector regained their visual responses to light with wavelengths between 468 and 640 nm and their recovered visual responses were maintained for a year. Thus, mVChR1 is a candidate gene for gene therapy for restoring vision, and gene delivery of mVChR1 may provide blind patients access to the majority of the visible light spectrum.

## Introduction

Channelrhodopsin-2 (ChR2) derived from the unicellular green algae *Chlamydomonas reinhardtii*^1,2^ has unique characteristics as a light-activated cation-selective channel.^3^ The structure of ChR2 is similar to that of bacteriorhodopsin,^4^ which includes a chromophore to absorb photons. Light causes the isomerization of the *all*-*trans* retinal chromophore to 13-*cis* retinal, which results in a conformational change. In the case of ChR2, the conformational change directly induces cation influx through the membrane.^3^ This specific feature allows us to generate photosensitive neurons by the transfer of a single gene, *ChR2*, to neuronal cells.^5,6^ The discovery of ChR2 and advances in techniques^6,7,8^ has introduced the field of optogenetics in neuroscience.

A new strategy for restoring vision includes the transduction of the ChR2 gene into retinal ganglion cells (RGCs)^9,10^ or ON-bipolar cells^11^ in genetically blind mice and rats. These animals are models for retinitis pigmentosa (RP),^12,13,14,15^ which is a retinal degenerative disease associated with a loss of photoreceptors, arising from mutations in genes related to the phototransduction pathway in the retina.^16^ This progressive photoreceptor degeneration finally leads to blindness. Gene therapy using ChR2 is a potential method for restoring vision to RP patients.^17^

The action spectrum of ChR2 peaks at 460 nm,^3^ which is similar to the spectrum for visually evoked potentials (VEPs) in ChR2-transduced genetically blind rats.^18^ Other ChRs such as *Volvox*-derived ChR1 (VChR1)^19^ have a broader, red-shifted action spectrum that is useful for restoring vision. Zhang *et al.* identified VChR1, whose action spectrum peaks at 535 nm, through Basic Local Alignment Search Tool searches. New channelrhodopsins with unique characteristics have been developed by modification of amino acids (aa) or generation of chimeric proteins with other channelrhodopsins; examples include a bistable step-function opsin that comprises sequences from ChR1 and VChR1.^20^ Use of an step-function opsin allows the photocurrent to be precisely initiated and terminated in response to different colors of light.^21^In the this study, we initially planned to use VChR1 to increase the wavelength sensitivity for restoring vision and therefore transferred the VChR1 gene into genetically blind rats by using an adeno-associated virus (AAV) vector. However, we could not record any VEPs in the VChR1-transferred blind rats and found that VChR1 had extremely low plasma membrane integration. To correct this deficiency, we identified a presumed signal peptide in the N terminal of ChR1 (aa1–23) by using the SOSUI software (http://bp.nuap.nagoya-u.ac.jp/sosui/)^22^ and generated a chimera of VChR1 and *Chlamydomonas* ChR1 (mVChR1). We restored vision for the entire visible light spectrum in genetically blind rats through mVChR1 transfer to RGCs.

## Results

### Expression profile of mVChR1

**Figure 1:**
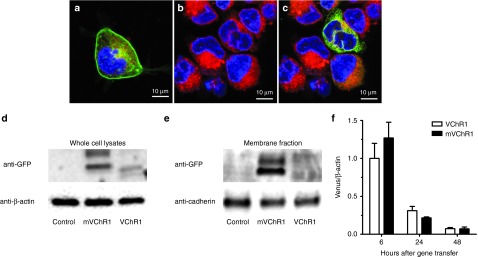
**Expression profiles of modified *Volvox* channelrhodopsin-1 (mVChR1) and *Volvox*-derived channelrhodopsin-1 (VChR1) in HEK 293 cells**. Fluorescence microphotographs of HEK 293 cells electroporated with plasmids expressing (**a**) mVChR1 or (**b**,**c**) VChR1. Photographs (**a**,**b**) were taken with same settings (laser intensity and exposure time). Photograph (**c**) was taken with increased exposure time to visualize VChR1 expression. Some of the fluorescent signals of the venus tag (green) were located in the endoplasmic reticulum (red). mVChR1 protein was clearly detected by western blotting in whole-cell lysates (**d**) and localized in the membrane fraction (**e**). Real-time polymerase chain reaction indicated no significant difference in mRNA expression (**f**). Data are shown as the mean ± SD values (*n* = 4). GFP, green fluorescent protein.

mVChR1 expression was clearly seen as Venus protein fluorescence in Human embryonic kidney (HEK)293 cells (**Figure 1a**). VChR1 expression (**Figure 1b**) was not observed under imaging with the same exposure time used for mVChR1. When the exposure time was increased, VChR1 expression was observed mainly in the cytoplasm and not in the plasma membrane (**Figure 1c**), which indicates that VChR1 expression was lower than mVChR1 expression. Western blot analysis using whole cell lysates (**Figure 1d**) or membrane fractions (**Figure 1e**) also showed abundant expression of mVChR1 in HEK293 cells, which was localized to the plasma membrane. The transduction efficiency can affect transgene expression. We investigated the mRNA expression of each gene by real-time polymerase chain reaction after electroporation; there was no significant difference in mRNA expression (**Figure 1f**).

### Patch clamp recordings

**Figure 2:**
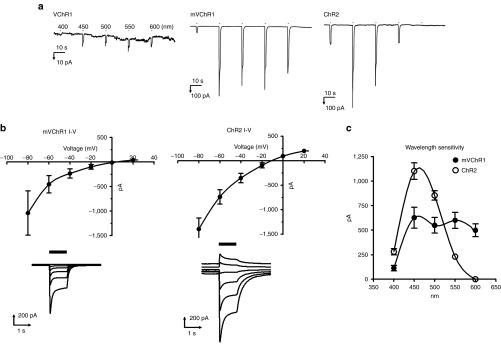
**Comparison of the photocurrent**. Typical waveforms of VChR1-, modified *Volvox* channelrhodopsin-1 (mVChR1)-, and *Chlamydomonas* channelrhodopsin-2 (ChR2)-expressing cells are shown in (**a**). For the I–V relationship analysis, stimulation at 500 ± 25 and 450 ± 25 nm was applied with a 1-second duration every 10 seconds for mVChR1- and ChR2-expressing cells (**b**), respectively. Each data point represents the mean ± SD (*n* = 6). Photocurrents induced by stimuli of various wavelengths are shown in (**c**). Each data point represents the mean ± SD value (*n* = 10). VChR1, *Volvox*-derived channelrhodopsin-1.

Under whole-cell patch clamp at −60 mV, VChR1- and mVChR1-expressing cells, but not ChR2-expressing cells, showed a photo-induced current upon stimulation even at 600 nm (**Figure 2a**). The evoked currents (626.3 ± 89.0 pA; 450 nm) in mVChR1-expressing cells were ~30 times larger than those (18.4 ± 22.3 pA; 450 nm) in VChR1-expressing cells. Investigation of I–V relationships showed that the photocurrent of mVChR1 was rectified to a lesser extent than that of ChR2 (**Figure 2b**). mVChR1-expressing cells had a broader action spectrum than ChR2-expressing cells (**Figure 2c**).

### Recording of visually evoked potentials

**Figure 3:**
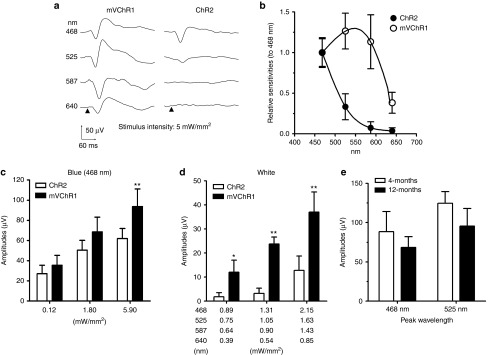
**Recordings of VEPs from modified *Volvox* channelrhodopsin-1 (mVChR1) or *Chlamydomonas* channelrhodopsin-2 (ChR2) gene-transferred rats**. Typical waveforms in response to light-emitting diodes (LEDs) of various wavelengths in mVChR1- and ChR2-transferred rats (**a**). Wavelength sensitivity is shown in (**b**). Recorded amplitudes were normalized against the amplitude of the response to 468-nm stimulation. Evoked potentials in response to blue LED (**c**) or white LED (d) stimulation are shown. Data (**b**–**d**) are shown as the mean ± SD values (*n* = 8).Changes in visually evoked potentials in mVChR1-transferred rats at 4 and 12 months after AAV administration are shown (**e**). Data are shown as the mean ± SD values (*n* = 4).

RGCs are functionally maintained in Royal College of Surgeons rats after photoreceptor degeneration although the number of RGCs decrease as they age.^23^ VEPs in photoreceptor-degenerated rats were not evoked even by the maximum intensity of light-emitting diode (LED) because of the loss of photoreceptors. VEPs in retinas transduced with the mVChR1 gene were clearly evoked upon LED stimulation between 468 and 640 nm, whereas VEPs were observed in retinas transduced with the ChR2 gene only in response to wavelengths lower than 525 nm (**Figure 3a**,**b**). Robust VEPs were evoked by blue LED stimulation in retinas transduced with the mVChR1 or ChR2 gene. The VEP amplitudes recorded for mVChR1 were slightly larger than those for ChR2 (**Figure 3c**). Notable differences were observed between mVChR1- and ChR2-transferred retinas with respect to the responsiveness to white LED stimulation (**Figure 3d**). Importantly, the recovered VEP amplitudes were maintained 12 months after AAV administration, although a tendency toward decreasing amplitudes (~25% reduction) with two wavelength stimuli was observed, which may indicate immunological tolerance (**Figure 3e**).

### Behavioral tests

**Figure 4:**
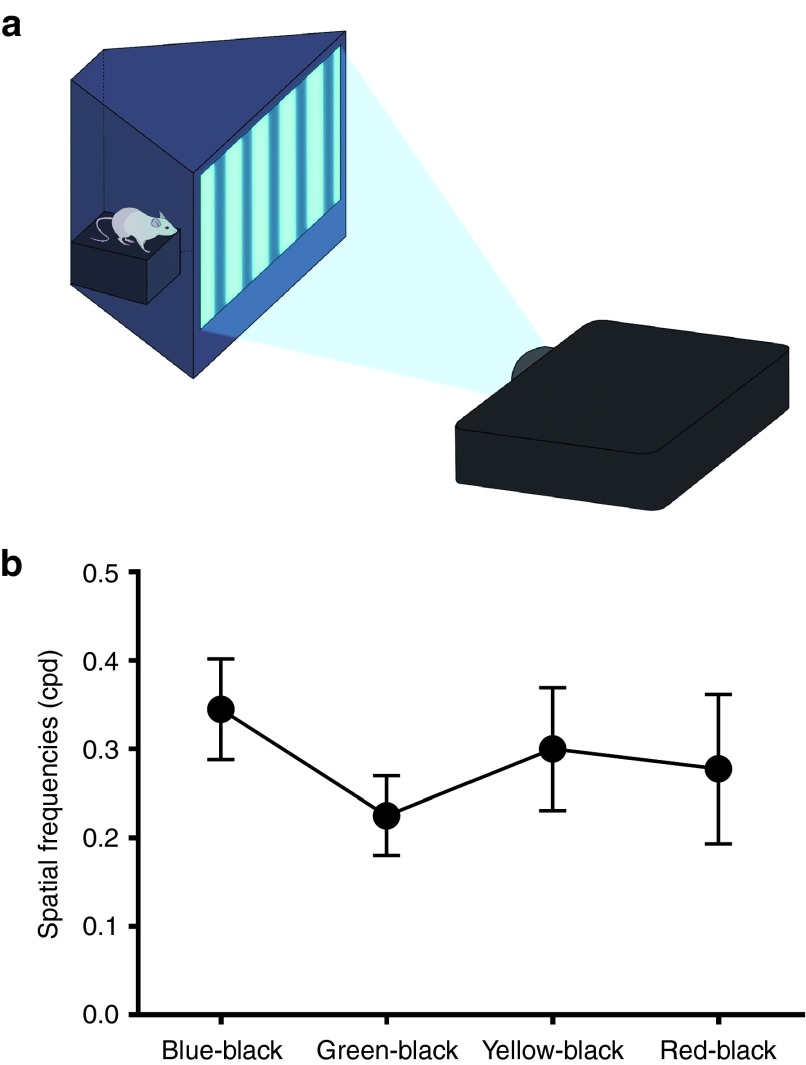
**Behavioral assessments in mVChR1 transduces Royal College of Surgeons rats**. Schematic representation of the optomotor apparatus. The grating pattern of blue, green, yellow, or red with various spatial frequencies (0.06, 0.09, 0.18, 0.36, 0.42, and 0.52) was projected onto a screen by a projector (**a**). The threshold spatial frequencies for the rats are shown in (**b**). Data represent the mean and the standard deviation (*n* = 8).

The spatial vision of an animal was quantified by its optomotor response. In our virtual optomotor system, a stimulus of color stripes over a black background was produced according to a sine wave function with variable frequency (**Figure 4a**). All of the rats tracked the objects when the spatial frequency was set at 0.18. However, the responses from all rats became undetectable when each color and black stripes were presented at 0.52. In the case of green–black stripes, no response was observed at a spatial frequency at 0.42. The mean values of spatial frequency showed less sensitivity to the green–black stripes than to stripes of other colors (**Figure 4b**).

### Expression profiles of channelrhodopsins in the retina

**Figure 5:**
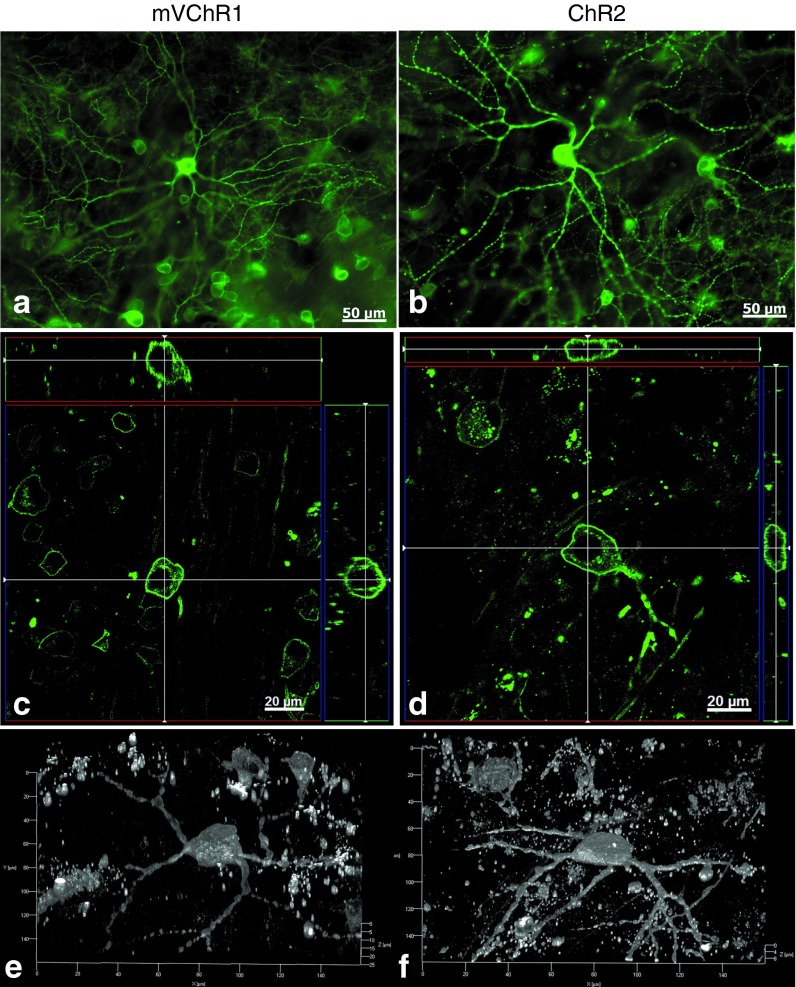
**Modified *Volvox* channelrhodopsin-1 (mVChR1) and *Chlamydomonas* channelrhodopsin-2 (ChR2) gene expression in whole-mounted retinas**. The expression of (**a**) mVChR1 and (**b**) ChR2 was clearly identified as Venus protein fluorescence in the plasma membrane (**c**,**d**). The 3D image stack shows that the expression of mVChR1 (**e**) in the dendriteswas lower than that of ChR2 (**f**).

mVChR1 and ChR2 were overexpressed in the retina and were mainly observed in RGCs (**Figure 5a**,**b**). mVChR1 (**Figure 5c**), and ChR2 (**Figure 5d**) expression was mainly seen at the membrane surface of the RGC soma. However, some differences were observed in the dendritic expression profiles of mVChR1 and ChR2. The 3D image stack clearly shows that the dendritic localization of mVChR1 was less than that of ChR2 (**Figure 5e**,**f**).

## Discussion

The discovery of ChR2 introduced optogenetics as a new field in neuroscience and provided a new strategy for restoring vision to blind patients. However, the visual function of ChR2 lacks sensitivity to wavelengths over 540 nm.^18^ VChR1 derived from *Volvoxcateri* can detect longer wavelengths than those for ChR2 and is thus another candidate gene for restoring vision. Here, we found that the VChR1 protein is mainly localized in the cytosolic fraction, which may be one of the reasons for its weak sensitivity to light. Modified VChR1 in which the N terminal fragment of VChR1 is replaced by the N terminal fragment of ChR1 of *Chlamydomonas*, which is highly homologous to the mammalian signal peptide for membrane translocation, demonstrated markedly increased membrane translocation and ion channel properties. VEPs in genetically blind rats that received mVChR1 could be recorded in response to light stimuli even at wavelengths >540 nm, and these rats showed higher sensitivity to white light stimuli than ChR2-transferred blind rats. Interestingly, the wavelengths sensed by mVChR1 overlapped those sensed by enhanced *Natronomonas pharaonis* halorhodopsin.^24,25^ Busskamp *et al.*^26^ reported that light-insensitive cones in a RP mouse model were reactivated by the expression of enhanced *N. pharaonis* halorhodopsin, and retinal cone pathways were restored. A combination treatment of enhanced *N. pharaonis* halorhodopsin and mVChR1, such as cone-specific enhanced *N. pharaonis* halorhodopsin transfer and peripheral ganglion cell-specific mVChR1 transfer, may therefore be useful for restoring highly organized vision in blind patients.

We had previously reported that chimeric proteins generated from multiple combinations of the ChR1 and ChR2 transmembrane helices from *Chlamydomonas* showed various ion channel properties.^27^ On further investigation, we found a presumed signal peptide at the N terminal of ChR1 (aa1–23) by using the SOSUI software (http://bp.nuap.nagoya-u.ac.jp/sosui/).^22^ Two approaches, *i.e.*, the window-based method^28^ and the global structure method,^29^ have been reported for the prediction of signal peptides. The disadvantage of the latter approach, which is based on the recognition of the three-domain structure of signal peptides, is its relatively low accuracy. The SOSUI method improves the accuracy of the global structure method by adding three modules (numeration of hydrophobic segments, prediction of signal sequences, and discrimination of signal peptides) to the software system.

VChR1 expression was observed in HEK293 cells when the plasmid vector including VChR1 was electroporated into cultured cells. However, obvious VEP responses were not recorded in VChR1-transferred rats. Over time, the number of VChR1-expressing cells decreased (see **Supplementary Figure S2**), and confocal microscopy indicated that the VChR1 protein was mainly localized to the cytoplasm but not the plasma membrane. Many studies have found that misfolded proteins have toxic effects on cells^30^ and can cause diseases.^31^ In most cases, misfolded proteins accumulate in the endoplasmic reticulum, which leads to a stress response^32^ that is thought to cause diseases, such as cystic fibrosis^33,34^ and RP.^35,36^ Therefore, it is possible that that cells expressing VChR1 die because of endoplasmic reticulum stress induced by misfolded VChR1. Importantly, with regard to mVChR1, the recovered visual responses have been maintained for a year since the AAV-mVChR1 was administered, although the recorded amplitudes declined slightly compared to those recorded at 4 months after gene transfer (**Figure 3e**). We have previously reported that no immunological responses were elicited by AAV-mediated ChR2 gene transfer into RGCs over the observation period (20 months).^37^ Doroudchi *et al.*^38^ have also shown that chronic ChR2 expression in the ON-bipolar cells of rd1 mice is nontoxic, with transgene biodistribution limited to the eye. The eye is considered to be an immunologically protected space.^39^ Therefore, the expression of channelrhodopsins should be well tolerated if it does not directly cause cytotoxicity. In addition, mVChR1 expression was clearly found in the retina and in the cell membrane of RGC somata (**Figure 5c**). The lower expression of mVChR1 than ChR2 in dendrites and axons in the retina may be beneficial because light perception by the axons may interfere with visual transmission.^40^

Kato *et al.*^41^ were the first to identify the crystal structure of the channelrhodopsin light-gated cation channel, and they showed that Asp 292 and Glu 136 in ChR1 are important for photocurrent and cation conduction, respectively. In mVChR1, these residues were well preserved, and Cys 166, Cys 197, and Ala 294 were also conserved from VChR1 because they may contribute to color shift. The peak wavelength of mVChR1-expressing HEK293 cells was ~530 nm, which coincides with the peak wavelength for VChR1 described in a previous study. However, another peak was observed at 470 nm. It is not clear why mVChR1 had a second peak that was not shown by VChR1, although it may have resulted from the replacement of the N terminal signal. In ChR1, aa1–23 were predicted to comprise a signal peptide. mVChR1 additionally includes aa24–66 of ChR1. Therefore, the presence of aa24–66 of ChR1in the N terminal of mVChR1 may affect the protein conformation of mVChR1 to produce another peak at 470 nm. In the behavioral tests, we also investigated the wavelength sensitivity in the rats transduced with the mVChR1 gene. The optomotor responses were elicited by stripes of all colors. We previously reported that the contrast sensitivity was low at a minimal spatial frequency of 0.06 cycles per degree (CPD), whereas it increased with an increase in spatial frequency, reaching a maximum at ~0.18 CPD, and was negligible at spatial frequencies over 0.52 CPD. The mVChR1-transduced rats responded to stripes of all colors at the spatial frequency of 0.18, at which the highest contrast sensitivity was shown in our previous study.^42^ Considering the human visual spectrum, the expansion of the wavelength sensitivity would be important to restore vision to patients with blindness.

In conclusion, the key findings of our study are that mVChR1 has broad-spectrum sensitivity and visual function *in vivo*. These results suggest that mVChR1 gene therapy may restore the majority of the visual spectrum for patients with RP through the transfer of only one gene. The resulting increased response to white light would also be helpful for the vision of patients who previously received channelrhodopsin gene therapy. We therefore believe that development of mVChR1 provides a critical step toward the restoration of normal vision in blind patients.

## Materials and Methods

***Animals.*** All experiments were conducted with the approval of the Animal Research Committee of the Graduate School of Medicine at Tohoku University. The experiments were conducted on 6-month-old male Royal College of Surgeons (rdy/rdy)^43^ rats obtained from CLEA Japan (Tokyo, Japan). The rats were kept under cyclic light conditions (12 hour on/off).

***Preparation of mVChR1.*** mVChR1 was designed as follows: The N terminal coding sequence (aa1–24) of VChR1 was replaced with that of ChR1 (aa1–67). mVChR1cDNA was subcloned into a 6P1-CAG plasmid (pAAVST)^9,10^ or pIRES vector (Takara, Shiga, Japan). (see **Supplementary Figure S1**).

***Expression profiles of VChR1- and mVChR1-expressing cells.*** The pAAVST plasmid, including VChR1 or mVChR1, was electroporated into cultured HEK293 cells. On the day after electroporation, the endoplasmic reticulum of the cells was stained with endoplasmic reticulum -Tracker Red (Life Technologies, Tokyo, Japan). VChR1 expression was observed using a confocal microscope (LSM710; Carl Zeiss, Tokyo, Japan). The membrane fractions of the cells were isolated using the ProteoExtract Native Membrane Protein Extraction Kit (Merck, Tokyo, Japan) to analyze the translocation of the protein to the cell membrane. Whole-cell lysates of gene-transduced cells were extracted using radioimmunoprecipitation assay buffer (5 mmol/l Tris–HCl (Wako Chemicals, Osaka, Japan)) (pH 7.6), 150 mmol/l NaCl (Wako Chemicals), 1% NP-40 (Sigma-Aldrich, Tokyo, Japan), 1% sodium deoxycholate (Sigma-Aldrich), 0.1% sodium dodecyl sulfate (Sigma-Aldrich), and protease-inhibitor cocktail (Roche Applied Science, Tokyo, Japan)). Membrane fractions (1.5 µg/lane) and whole-cell extracts (30 µg/lane) were electrophoresed on 4–15% sodium dodecyl sulfate polyacrylamide gels (Mini-PROTEAN TGX gel; Bio-Rad Laboratories, Tokyo, Japan) and transferred onto a polyvinylidene difluoride membrane (Bio-Rad Laboratories). The membranes were bathed with an anti-green fluorescent protein antibody (no. 598, MBL; Nagoya, Japan), which was crossreactive with the Venus protein, an anti-β-actin antibody (sc-69879; Santa Cruz Biotechnology, Dallas, TX), or an antipan cadherin antibody (sc-59876; Santa Cruz Biotechnology) and then washed three times with Tris-buffered saline with Tween 20 (10 mmol/l Tris–HCl (pH 8.0), 150 mmol/l NaCl, and 0.1% Tween 20). Alkaline phosphatase-conjugated goat antirabbit or antimouse IgG (Promega, Tokyo, Japan) was used as the secondary antibody. Protein bands were developed using CDP Star (Roche Applied Science) according to the manufacturer's instructions.

***Semiquantitative real-time polymerase chain reaction.*** Total RNA was isolated from the cultured HEK 293 cells by using the Absolute RNA nanoprep kit (Agilent Technologies, Santa Clara, CA) at 6, 24, and 48 hours after cells were electroporated with plasmid DNA, including VChR1- or HmVChR1-Venus cDNA. cDNA was synthesized using ReverTra AceqPCR RT Master Mix (Toyobo, Osaka, Japan). The sense and antisense primers for venus were 5′-CTATATCACCGCCGACAAGC-3′ and 5′-GGGGTGTTCTGCTGGTAGTG-3′ and those for β-actin were 5′-CTGGAACGGTGAAGGTGACA-3′ and 5′-AAGGGACTTCCTGTAACAATGCA-3′. The expression level of each gene was calculated after normalization to β-actin. SYBR Green Supermix (Bio-Rad Laboratories, Tokyo, Japan) was used for real-time polymerase chain reaction, and the specific transcripts were amplified using CFX Connect (Bio-Rad laboratories, Hercules, CA).

***Establishment of stable transformants expressing the mVChR1 gene.*** HEK293 cells were cultured in Dulbecco's modified Eagle medium (Life Technologies) supplemented with 10% fetal bovine serum under a 5% CO_2_ atmosphere at 37 °C. The expression plasmid (pmVChR1-IRES-puro, pVChR1-IRES-puro, or pChR2-IRES-puro) was linearized using a restriction enzyme and electroporated into cultured HEK293 cells using the CUY21Pro-vitro system (Nepa Gene, Chiba, Japan). Transformants were selected in culture medium containing puromycin (0.1–30 µg/ml) for at least 10 days.

***Patch clamp recordings.*** Photocurrents were recorded using an EPC-10 amplifier (HEKA Electronic, Lambrecht, Germany) under whole-cell patch clamping of isolated cells. Series resistance was compensated up to 70% to reduce series resistance errors. The data were collected by filtering at 10 kHz and sampled at 20 kHz. The internal solution contained 120 mmol/l CsOH, 100 mmol/l glutamate, 50 mmol/l 4-(2-hydroxyethyl)-1-piperazineethanesulfonic acid (HEPES, Sigma-Aldrich), 2.5 mmol/l MgCl_2_, 2.5 mmol/l MgATP, 5 mmol/l Na_2_EGTA, and 1.2 mmol/l leupeptin, with the pH adjusted to 7.2 for whole-cell current recordings. Tyrode's solution contained 134 mmol/l NaCl, 3mmol/l KCl, 2.5 mmol/l CaCl_2_, 1.25 mmol/l MgCl_2_, 4mmol/l NaOH, 10 mmol/l HEPES (Sigma-Aldrich), and 2 g/l glucose, with the pH adjusted to 7.4 by HCl. Photostimulation was performed on an inverted microscope (Eclipse; Nikon, Tokyo, Japan) equipped with a xenon lamp and electromagnetic shutter (Unibilitz, Rochester, NY). Various wavelengths (400, 450, 500, 550, and 600 nm) of light were produced by setting a band-pass filter (Fujifilm, Tokyo, Japan) in the carousel of the inverted microscope. The intensity of light at each wavelength was also adjusted to 0.25 mW/mm^2^ by setting an appropriate density filter for each wavelength into the carousel. The photocurrent was measured by two to three repetitions of a protocol, in which the wavelength was changed from 400 to 600 nm and the reverse order was applied with 1 second of light exposure. For the I–V relationship analysis, stimulation at 500 ± 25 and 450 ± 25 nm was applied with a 1-second duration every 10 seconds for mVChR1- and ChR2-expressing cells, respectively.

***Preparation of the AAV vector.*** Plasmid vectors containing ChR2, VChR1, or mVChR1 were used for production of the AAV vector. The basic construction of the plasmid vector has been previously described.^9,10^ The AAV Helper-Free System (Agilent Technologies) was used to produce infectious AAV virions (AAV-ChR2V, AAV-VChR1V, and AAV-mVChR1V) according to the manufacturer's instructions. AAV vectors were purified by a slight modification of a previously described single-step column purification method.^44^ The concentration of the purified AAV vectors was determined by measuring AAV capsid protein levels using ELISA.

***Intravitreal injection of AAV.*** Rats were anesthetized by intramuscular injection of 66 mg/kg ketamine and 33 mg/kg xylazine. Then, using an operating microscope, an incision was made in the conjunctiva to expose the sclera. Five microliters of a suspension that contained 1–10 × 10^12^ virions⋅µ/l was intravitreally injected through the ora serrata by using a 10-µl Hamilton syringe with a 32-gauge needle (Hamilton Company, Reno, NV).

***Recording of visually evoked potentials.*** Four months after the intravitreous injections, VEPs were recorded as described previously.^17,18,23,37^ Briefly, VEPs were recorded using a Neuropack (MEB-9102; Nihon Kohden, Tokyo, Japan). First, the rats were anesthetized with ketamine–xylazine, and then their pupils were dilated with 1% atropine and 2.5% phenylephrine hydrochloride. Photic stimuli of various intensities were generated from different colors of LEDs and applied for 10 ms with a frequency of 0.5 Hz. The high- and low-pass filters were set to 50 and 0.05 kHz, respectively. VEP responses were measured 100 consecutive times, and then the response waveform was averaged. To investigate the spectrum sensitivity, the intensity of each light was adjusted to 5 mW/mm^2^ by controlling the voltage used to activate the LEDs. The *x* axis in **Figure 3d** indicates the light intensity of the white LED corresponding to each wavelength.

***Behavioral tests.*** We used a virtual optomotor system to evaluate the optomotor responses. The original virtual optomotor system described by Prusky *et al*.^23^ was modified for rats.^42^ A light–dark grating pattern was projected onto a screen in front of a platform by using a projector (MX764; BenQ Japan, Tokyo, Japan) (**Figure 4a**). A video camera was stationed 50 cm above the platform to record the rat's movements and the screen. The grating patterns were determined by a sine wave function with variable spatial frequency. The virtual rotation speed was set at 12 degrees per second (2 rpm) in all experiments. The maximum intensity reached 2,500 lux of white light at the platform. However, the intensity varied according to the color of the light stripes presented. The luminance at the center of the platform was 140, 800, 1,550, 200, and 1,600 lux when the color was set to blue, green, yellow, red, and gray, respectively. Each wavelength projected on the screen was measured by using a spectroscope (C1083CAH; Hamamatsu Photonics, Shizuoka, Japan). The rat was allowed to move freely on the platform in the virtual optomotor system. The experimenter waited until the rat stopped moving, and then a homogeneous gray stimulus was projected for 20 seconds on the screen before the presentation of each grating session for 60 seconds. The grating session was started from a low spatial frequency (0.06 CPD). An experimenter assessed whether the animals tracked the rotation by monitoring the head movement and the presented rotating stimulus simultaneously on the video camera monitor. If head movement occurred simultaneously with the rotation, the experimenter judged that the animal could discriminate the grating and proceeded to the next grating session. If the movement was ambiguous, the same grating session was presented again. All behavioral tests were double blind and performed during the first few hours of the animals' light cycle (lights on at 8 AM).

***Expression profiles of channelrhodopsins in the retina.*** The animals were sacrificed by administering an overdose of intraperitoneal pentobarbital (Kyoritsu Seiyaku, Tokyo, Japan). The eyes were removed and fixed with 4% paraformaldehyde in 0.1 mol/l phosphate-buffered saline overnight at 4 °C, and the retinas were flat-mounted. The flat-mounted retinas were covered with Vectashield medium (Vector Laboratories, Burlingame, CA), and Z stacked images were captured at an interval of 0.38 µm under a confocal microscope (Carl Zeiss), followed by 3D image construction.

***Statistical analysis.*** Statistical analysis was performed using the GraphPad Prism software (GraphPad Software, San Diego, CA). The criterion for statistical significance was *P* < 0.05, and the unpaired *t*-test was used.

**SUPPLEMENTARY MATERIAL** **Figure S1.** Preparation of mVChR1. **Figure S2.** Changes in the positive cell number after VChR1 or mVChR1 gene transfer.
