# Supplementary figures and images for: Restoration of the Majority of the Visual Spectrum by Using Modified Volvox Channelrhodopsin-1
Source: Mol Ther. 2014 Jun 3;22(8):1434–40. doi: 10.1038/mt.2014.81 (PMC4435592; doi:10.1038/mt.2014.81)

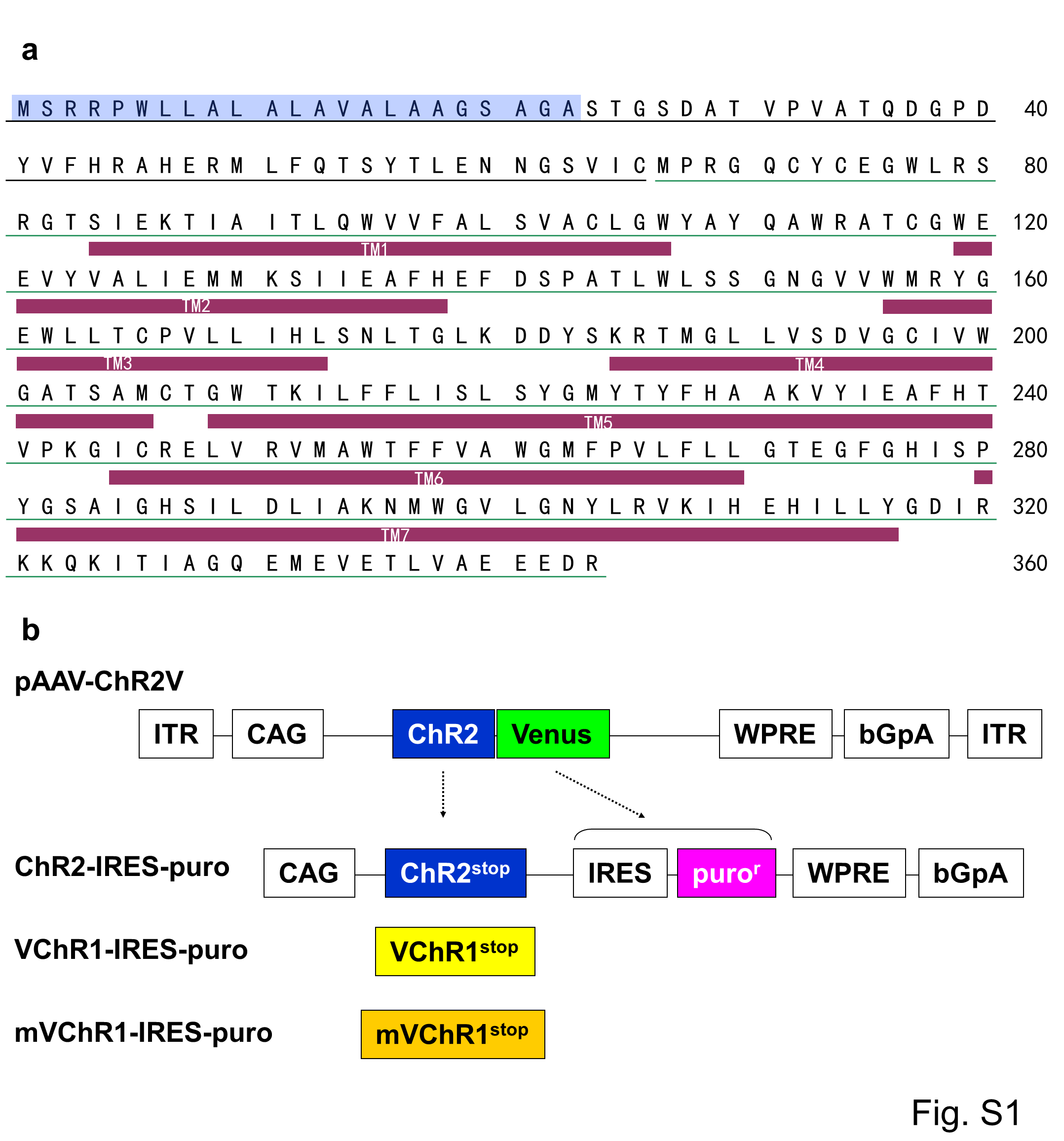

Supplement: Supplementary Figure S1 [file mt201481x1.tiff]

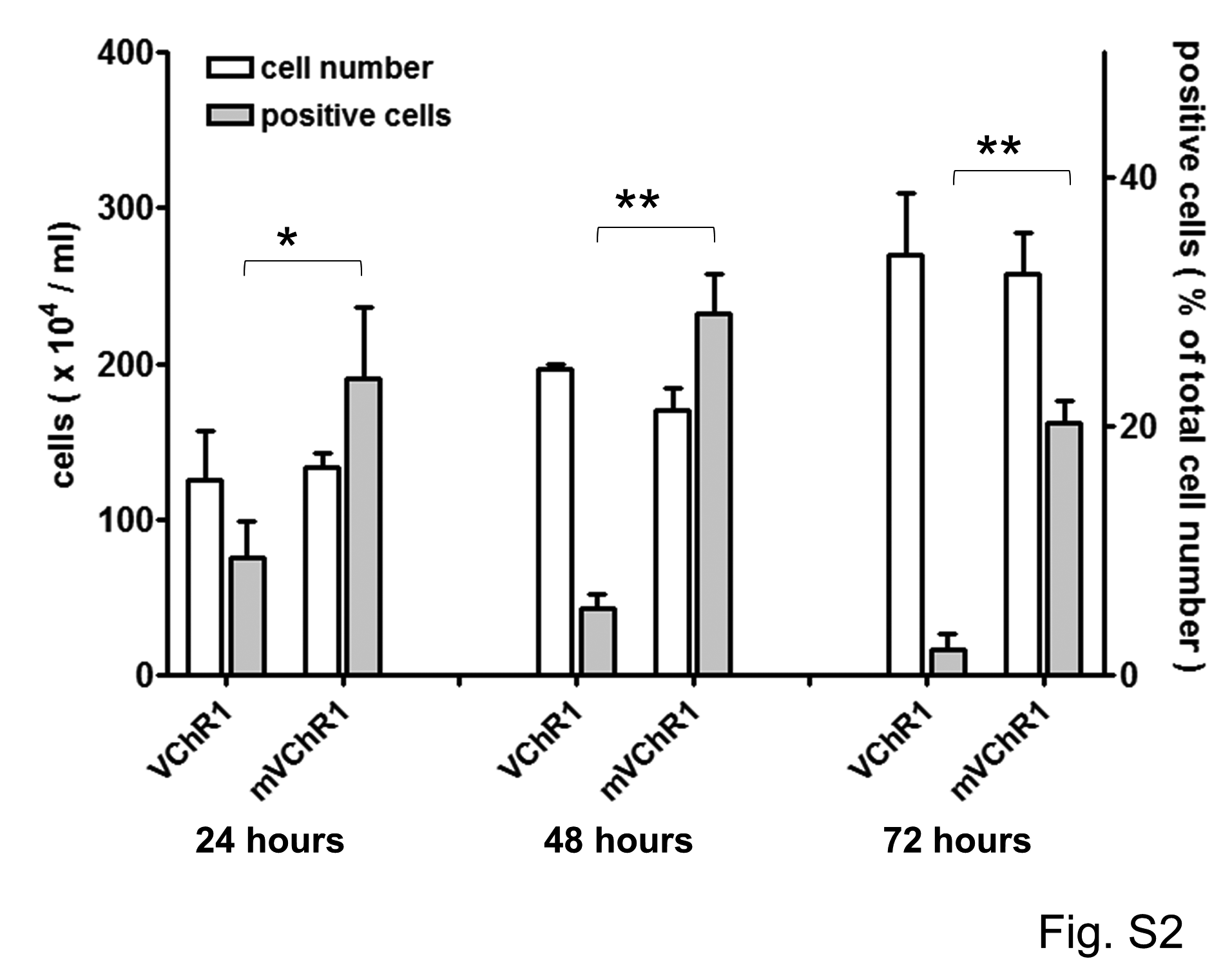

Supplement: Supplementary Figure S2 [file mt201481x2.tiff]
